# Simultaneously enhanced mechanical properties and flame retardancy of UHMWPE with polydopamine-coated expandable graphite

**DOI:** 10.1039/c9ra02861g

**Published:** 2019-07-09

**Authors:** Huaming Wang, Jingshi Cao, Fubin Luo, Changlin Cao, Qingrong Qian, Baoquan Huang, Liren Xiao, Qinghua Chen

**Affiliations:** College of Environmental Science and Engineering, Fujian Normal University China qrqian@fjnu.edu.cn cqhuar@fjnu.edu.cn; Fujian Key Laboratory of Pollution Control & Resource Reuse China; Engineering Research Center of Polymer Green Recycling of Ministry of Education Fuzhou 350007 China

## Abstract

The potential prospect of expandable graphite (EG) in the development of polymer composites is severely limited by required large additions and poor interface compatibility with the polymer. Inspired by mussels, polydopamine (PDA) can be used as an effective interface modifier for EG to prepare ultra high molecular weight polyethylene (UHMWPE) composites with superior mechanical properties and high flame retardancy. The surface of expandable graphite (EG) was coated with a thin adhesive PDA film through self-polymerization of dopamine. The modified expandable graphite (EG@PDA) was combined with APP to prepare UHMWPE flame retardant composites. Compared with UHMWPE/APP/EG (with 20 wt% APP/EG), UHMWPE/APP/EG@PDA (with 20 wt% APP/EG@PDA) gives a decrement by 16.7% in limiting oxygen index, 29.7% in the peak of the heat release rate, 20.4% in total heat release and 49.3% in total smoke release, with an increment by 37% in tensile strength and 67.9% in elongation at break, respectively. It is suggested that the presence of PDA as an interface modifier can greatly improve the interfacial compatibility between EG and UHMWPE. Moreover, it can lead to forming more char residue and reducing the release of smoke particulates during combustion of the composites.

## Abstract 2

The potential prospect of expandable graphite (EG) in the development of polymer composites is severely limited by required large additions and poor interface compatibility with the polymer.

## Graphical abstract

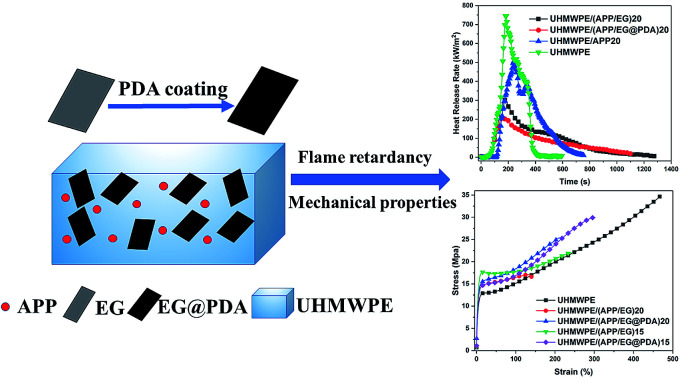

## 1. Introduction

With the wide application of flame-retardant composite materials, researchers are paying more and more attention to the mechanical properties of materials while pursuing flame retardant properties.^1^ How to prepare multifunctional flame-retardant materials without reducing their other excellent properties is a hot research topic.^2^ Ultra-high-molecular-weight polyethylene (UHMWPE) is called a special engineering plastic with remarkable performance.^3–5^ It is widely used in the military industry, transportation, and the textile and cable industry, which need extremely high flame-retardant performance.^6^ As we know, expandable graphite (EG)^7^ and ammonium polyphosphate (APP)^8^ have become important flame retardants instead of halogen-included flame retardants for twenty years due to their benefits to the environment.^9^ However, the large addition of halogen-free flame retardants caused the deterioration of mechanical properties of composites.^10,11^ In order to solve these problems, the interface modifier which simultaneously maintains the flame retardancy and mechanical properties has been studied for many years. The phosphorus flame retardant to modify EG for improving flame retardant effectiveness has been reported.^12^ It also has been reported that low-sulfur EG was prepared through a two-step intercalation method using environmentally friendly hydrogen peroxide as an oxidant.^13^ Though the flame-retardant efficiency has been improved *via* these approaches, the mechanical properties of polymers improve only a little, which extremely limits the application of polymer.

Polydopamine (PDA) is a mussel-inspired bio-polymer which was discovered by Lee *et al.* in 2007.^14^ Mussels can strongly attach to diverse substrates with high binding strength, even on wet surfaces. This phenomenon can be explained by the presence of PDA. Because of its special characteristic, PDA is widely studied and applied in many kinds of industrial and academic areas. For example, Lee *et al.* reported that bioinspired polymerization of dopamine to generate melanin-like nanoparticles has an excellent free-radical-scavenging property.^15^ Zhang *et al.* reported that raw flax fiber was coated with a thin adhesive polydopamine (PDA) film to added into PLA to prepare flame retardant composites.^16^ As reported in recent literatures, PDA can not only be a strong interface bridge to increase compatibility between filler and polymer,^17^ but also endow the polymer superior flame retardancy.^18,19^ Moreover, due to the similar molecular structure with mussels, it can be easily deposited on almost all types of inorganic and organic substrate.^20^ Based on the above discussion, PDA is capable of coating on the surface of EG and providing the composites excellent mechanical properties and flame retardancy.

Considering all of those aspects, PDA is used as a novel interface modifier to coat onto the surface of EG. PDA can significantly improve the mechanical properties and property of smoke suppression. The modification process was proposed through a sol–gel and surface treatment technologies which the prepared method is mild, environment friendly and economy. The structure of the resultant polydopamine-modified expandable graphite (EG@PDA) was characterized by Fourier transform infrared (FTIR), energy dispersive spectrometer (EDS) and scanning electron microscopy (SEM). The resultant EG@PDA was applied as a flame retardant and interface modifier for UHMWPE. After the introduction of EG@PDA into UHMWPE/APP, its mechanical properties, flame retardancy and smoke suppression of UHMWPE composites could be effectively enhanced. The thermal stability, flammability and mechanical properties of UHMWPE/APP/EG@PDA were investigated by thermogravimetry analysis (TGA), limiting oxygen index (LOI) determination, vertical burning test (UL-94), cone calorimeter test (CCT) and tensile test, respectively.

## 2. Experimental

### 2.1. Materials

UHMWPE powder with an average diameter of 150 μm (*ρ* = 0.93 g cm^−3^, *M* = 2.5 × 10^6^ g mol^−1^) was purchased from Lianle Chemical Co., Ltd. (Shanghai, China). Expandable graphite (EG) with particle size of 300 μm was supplied by Qingdao Kangboer Graphite Company. Dopamine hydrochloride and tris(hydroxymethyl)aminomethane (Tris) were purchased from Aladdin. All other chemicals and reagents were purchased from Beijing Chemical Works (China) and used as received.

### 2.2. Preparation of polydopamine coated expandable graphite (EG@PDA)

**Scheme 1:**
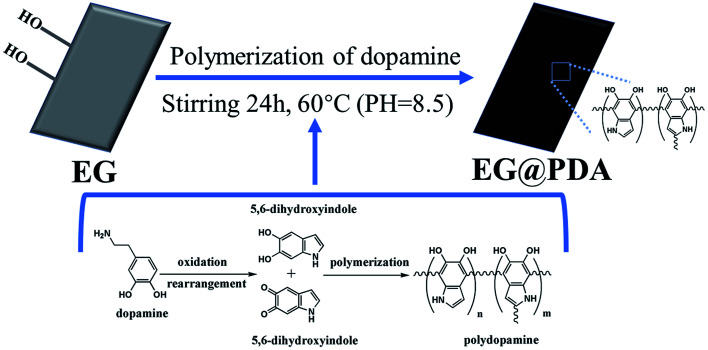
Schematic illustration of the preparation of the EG@PDA.

EG was washed with acetone, rinsed with distilled water several times followed by drying at 60 °C for 12 h. Then the dried 120 g EG was mixed with 2 g L^−1^ dopamine solution, which was prepared by dissolving dopamine hydrochloride in Tris–HCl buffer solution. The suspension was stirred at 60 °C for 24 h. Then the suspension was filtered and the resultant EG@PDA was washed with distilled water and dried at 45 °C overnight. The resultant is weight about 124.68 g which grafting rate is 3.9%. The preparation process of EG@PDA is shown in Scheme 1.

### 2.3. Preparation of UHMWPE composites

The nascent UHMWPE powder and flame retardants were premixed in a crusher (20 000 rpm) for 30 s. The mixture was hot compression molded on a flat vulcanizing machine (ZG-80T, made in Dongguan, China) at 200 °C for 10 min followed by cold compression molded to sheets at room temperature for 10 min at a pressure of 17 MPa. The formulations of the UHMWPE composites were presented in Table 1.

**Table 1:** Formulations of pure UHMWPE and its flame-retardant composites

Samples	UHMWPE (wt%)	EG@PDA (wt%)	EG (wt%)	APP (wt%)
UHMWPE	100	0	0	0
UHMWPE/(EG@PDA/APP)20	80	13.33	0	6.66
UHMWPE/(EG/APP)20	80	0	13.33	6.66
UHMWPE/APP20	80	0	0	20

### 2.4. Measurements and characterization

Fourier transform infrared spectra (FTIR) of EG and EG@PDA were tested on a Nicolet 560 spectrophotometer. FTIR spectrometer to detect the chemical structure of the EG and EG@PDA were ground with KBr into fine powders and then pressed the homogeneous mixture into a disk. FTIR spectra within the wave number range of 400–4000 cm^−1^ were obtained by averaging 16 scans at a resolution of 4 cm^−1^.

The LOI test was used to measure the minimum oxygen concentration to support candle-like combustion of samples. It was measured with specimens of 120 × 6.5 × 3.2 mm^3^ according to the ASTMD 2863-97 standard.

The Underwriters Laboratories-94 (UL-94) vertical burning test was carried out on a CZF-1 type instrument (Nanjing Jiangning Analytical Instrument Factory, China), with the bar dimensions of 125 × 13 × 3.2 mm^3^ according to ASTM D635-77.

Thermogravimetric Analysis (TGA) was used TA Instruments Q50 (TA Instruments Inc., USA) to determine EG, EG@PDA and composites, which were heated under nitrogen from 100 °C to 600 °C at a heating rate of 10 °C min^−1^ and the nitrogen flow rate of 90 mL min^−1^.

The surface morphologies and Energy Dispersive Spectrometer (EDS) study of the EG, EG@PDA, the char residues of various UHMWPE composites were observed on a scanning electron microscope (SEM) (Model JSM-7500F, Japan) with a conductive gold coating and with an acceleration voltage of 5.0 kV.

The tensile strength of composites was measured on a mechanical testing machine (W2Y-240, SANS) at ambient temperature according to GB 1040.1-2006 (cross-head speed 50 mm min^−1^).

The cone calorimeter (FTT0007, Fire Testing Technology, UK) tests were performed according to ISO 5660 standard procedures, with 100 mm × 100 mm × 3.2 mm specimens. Each specimen was wrapped in an aluminum foil and exposed horizontally to 35 kW m^−2^ external heat flux.

## 3. Results and discussion

### 3.1. The synthesis and characterization of EG@PDA

The mechanism for synthesis of EG@PDA are illustrated in Scheme 1. It is suggested that the surface of EG@PDA covered by polydopamine which formed by self-polymerization of dopamine. After reaction of polymerization, EG@PDA was obtained with a heavy black colour which indicates that EG are well-coated in dopamine solution. The mechanism of polymerization of dopamine may be explained by two steps: one is dopamine monomers are believed to form 5,6-dihydroxyanthraquinone through the oxidation and rearrangement of dopamine molecules. The other is that 5,6-dihydroxyanthraquinone is unstable, easily polymerizes to form polydopamine which produced by an intermolecular cross-linking reaction.^21–23^

**Fig. 1:**
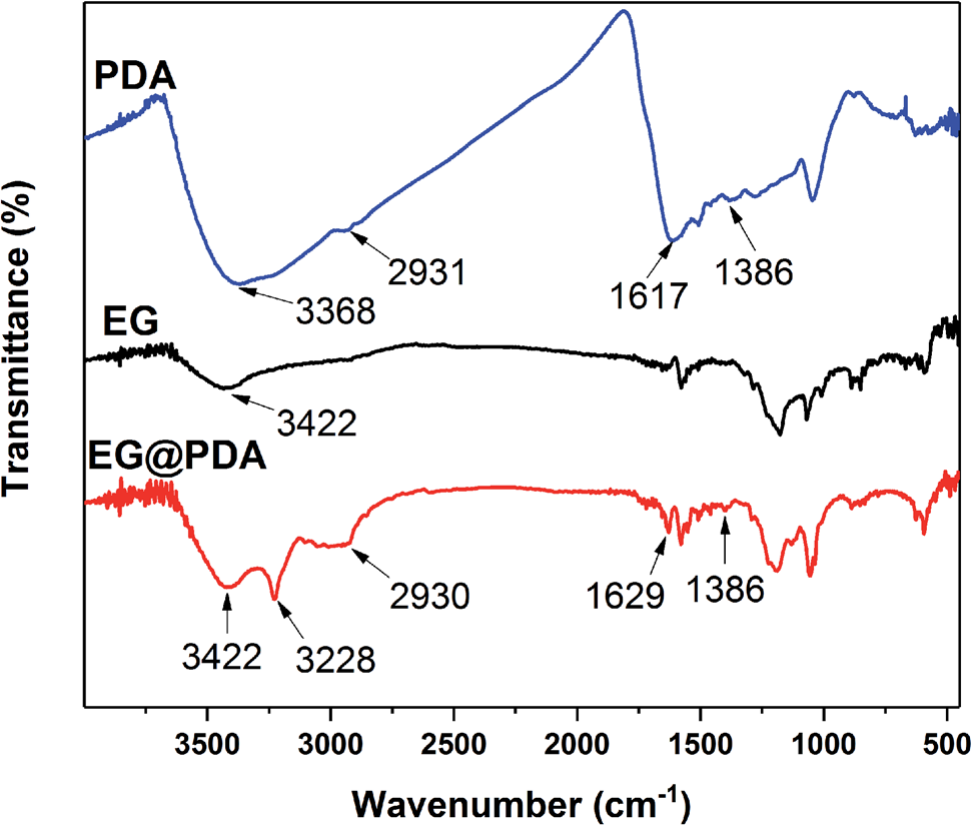
FTIR spectra of EG and EG@PDA.

FTIR spectra for EG and EG@PDA are presented in Fig. 1 and Table 2. For EG, the peak at 3422 cm^−1^ is attributed to O–H stretching vibrations. The O–H may be introduced through acid oxidation treatment in the production process.^24^ For PDA, these character peaks at 3368, 2931, 1617 and 1386 cm^−1^ are associated with the O–H stretching vibrations, the stretching vibration of alkyl group, C=C stretching vibrations in the aromatic ring and the bending vibration of phenolic C–O–H, respectively.^25,26^ For EG@PDA, these peaks at 3422, 2930, 1629 and 1386 cm^−1^ are as same as the characteristic peaks of PDA. In addition, a new peak at 3228 cm^−1^ assigns to hydrogen bond suggests that the method of PDA coated EG maybe through the hydrogen bond. These results indicate that PDA was successfully coated on the surface of EG.

**Table 2:** Assignments of the peaks in FTIR spectra

Wavenumbers (cm^−1^)	Assignment
3422, 3368	Stretching vibration of O–H groups
3228	Hydrogen bond
2931, 2930	C–H stretching vibration in –CH_3_
1629, 1617	C=C stretching vibrations in the aromatic ring
1386	The bending vibration of phenolic C–O–H

**Fig. 2:**
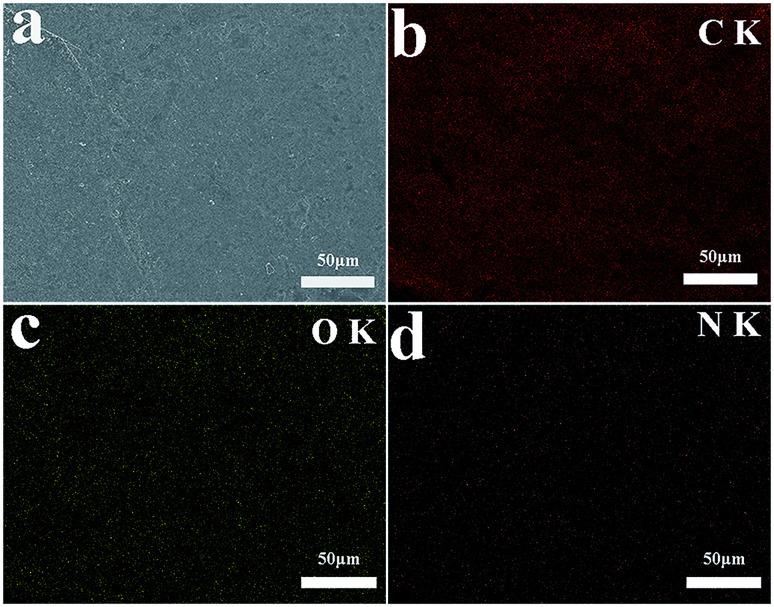
The surface morphology and EDS elemental data of EG (a and c) and EG@PDA (b and d).

**Fig. 3:**
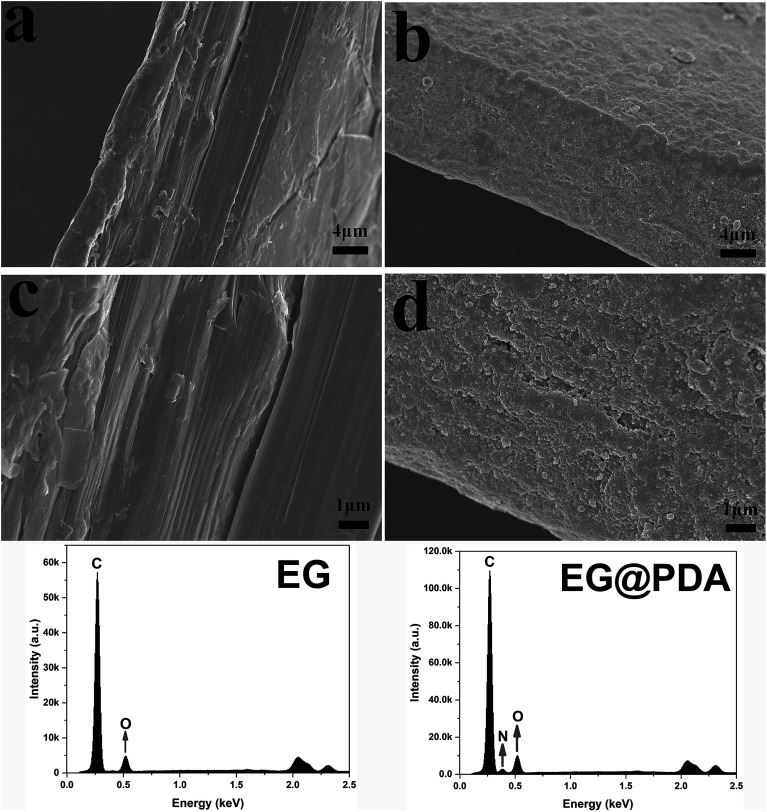
SEM image of EG@PDA (a) and EDS elemental mapping of EG@PDA: carbon element (b), oxygen element (c) and nitrogen element (d).

SEM analysis can be used to study the surface morphology of particles to confirm the formation of polydopamine in EG@PDA (Fig. 2).^27^Fig. 2a and c are photos of the side surface of EG, on where the graphite sheet layers are clearly observed. However, the graphite sheet layers cannot be observed on the side surface of EG@PDA in Fig. 2b and d instead of much small particles cover on the surface of EG. In addition, the EDS analysis of EG@PDA shows that N element was found on the EG@PDA compared to EG, indicating the occurrence of PDA on the EG surface. Moreover, EDS mapping was carried out in the Fig. 3 and the calculated data was showed in the Table 3. Compared with EG, 3.49% weight percent N element and higher weight percent O element were found on EG@PDA, indicating that PDA was successfully coated on EG.

**Table 3:** EDS analysis results of EG and EG@PDA

Sample	Elements	Weight (%)	Atomic (%)
EG	C	85.59	88.78
	O	14.41	11.22
EG@PDA	C	81.47	85.08
	O	15.04	11.79
	N	3.49	3.13

**Fig. 4:**
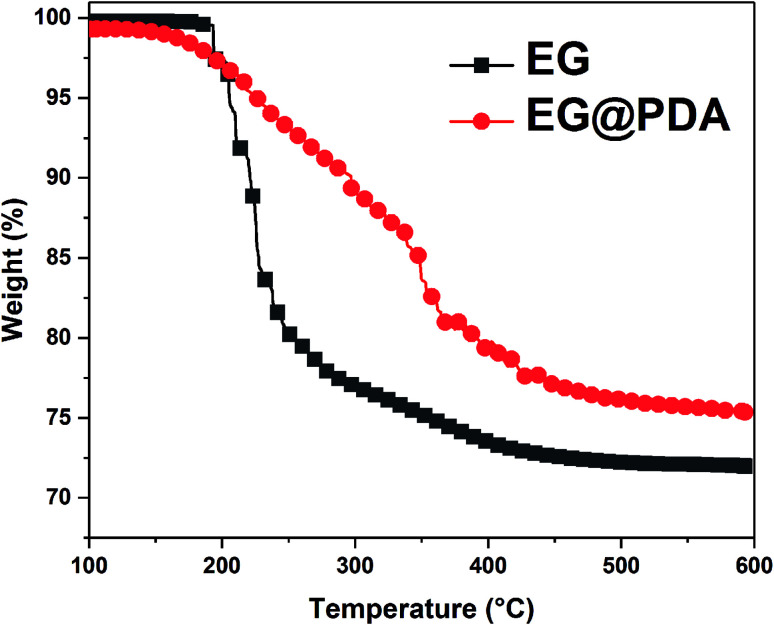
TGA curves of EG and EG@PDA in N_2_ atmosphere.

The TGA curves of EG and EG@PDA in N_2_ atmosphere are shown in Fig. 4. The temperatures at the relative different mass loss of 5% (*T*_5%_), 30% (*T*_30%_) and 50% (*T*_50%_) are listed in Table 4. It is clear that EG@PDA give a much higher TGA curve in shape compared to EG, indicating that the introduction of PDA onto the surface of EG leads to large improvement in its thermal stability.^28^ The residue of 75.3% for EG@PDA is higher than that of 72% for EG at 600 °C because of the presence of PDA which decompose at high temperature. These results indicate that PDA was successfully coated on EG.

**Table 4:** TGA data of UHMWPE and its flame-retardant composites^a^

Samples	*T*_5%_ (°C)	*T*_30%_ (°C)	*T*_50%_ (°C)	*T*_p_ (°C)	Residues at 600 °C (wt%)
EG	205.5	—	—	—	72
EG@PDA	226.2	—	—	—	75.3
UHMWPE	448.4	471.4	478.2	484.2	0
UHMWPE/(APP/EG)20	439.8	475.1	483.6	487.4	12
UHMWPE/(APP/EG@PDA)20	427.8	467.7	476.6	480.6	13.1
UHMWPE/APP20	449.6	474.9	482	484.9	10.6

a *T*_5%_, *T*_30%_, and *T*_50%_, onset decomposition temperature of 5%, 30%, and 50% weight loss, respectively; *T*_p_, peak temperature of DTG.

### 3.2. The flame-retardant performances of UHMWPE composites

Limiting oxygen index (LOI) and UL-94 vertical burning tests for UHMWPE and diverse UHMWPE composites were characterized and shown in Table 5. It was observed that UHMWPE exhibited high flammability that LOI value was as low as 17.5%, and there was no classification in UL-94 test. The introduction of flame retardant could notably improve LOI value of UHMWPE and enhance the flame retardancy of composites. It is noteworthy that the flame retardancy of UHMWPE/(APP/EG@PDA)20 has reached V-0 classification in UL-94 vertical burning test. The experiments were also implemented on UHMWPE/(APP/EG)20 and UHMWPE/(APP/EG)25. It is clear that UHMWPE/(APP/EG)20 give its LOI values of 26.2% with no classification of UL-94, whereas UHMWPE/(APP/EG)25 give the LOI value of 30.4% with V-0 classification in UL-94, suggesting that only large addition of APP/EG could obtain significant effects on the LOI value and UL-94 rating of UHMWPE. Moreover, UHMWPE/APP20 fails in the UL-94 test and reaches the LOI value of 22.1% compared with UHMWPE/(APP/EG)20, showed that APP and EG had remarkable synergistic effect in flame retardant UHMWPE, but still need plenty amount of APP/EG to achieve V-0 classification in UL-94 test. Compared with UHMWPE/(APP/EG)20, UHMWPE/(APP/EG@PDA)20 shows superior flame retardancy and reaches V-0 rating in UL-94 test. It is suggested that, the presence of PDA on the surface of EG could improve the flame retardant performance of UHMWPE composites.

**Table 5:** Formulations and flammability of pure UHMWPE and its flame-retardant composites

Samples	UL-94	LOI (%)
UHMWPE	NR	17.5
UHMWPE/(APP/EG@PDA)20	V-0	29.2
UHMWPE/(APP/EG)20	NR	26.2
UHMWPE/(APP/EG)25	V-0	31.8
UHMWPE/APP20	NR	22.1
UHMWPE/(APP/EG)15	NR	23.3
UHMWPE/(APP/EG@PDA)15	NR	25.9

**Fig. 5:**
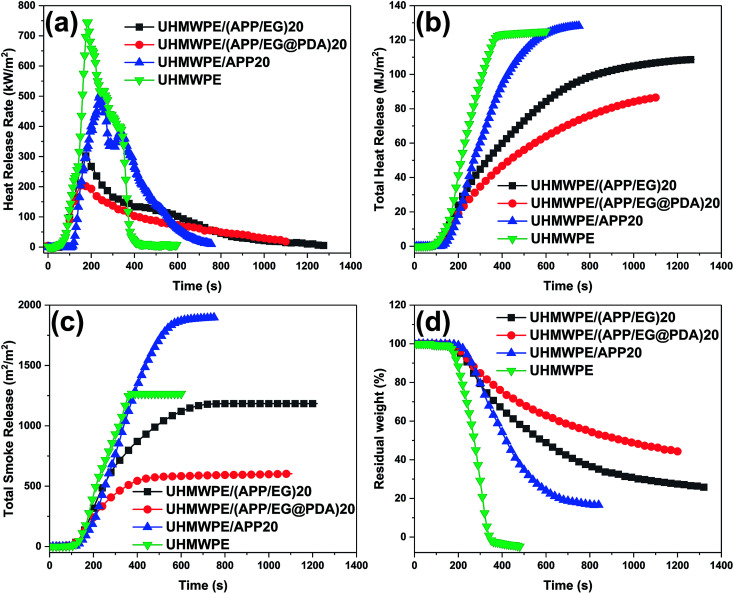
(a) HRR, (b) THR, (c) TSR and (d) residual weight for UHMWPE and UHMWPE composites.

The cone calorimeter is an important factor provides useful information about the burning behavior.^29^Fig. 5 shows the cone calorimetry analyses of UHMWPE and its composites. And the typical data are also summarized in Table 6. The time to ignition (*t*_ign_), time to PHRR (*t*_PHRR_), peak heat release rate (PHRR), total heat release (THR), total smoke release (TSR) and residual weight (%) are six important parameters representing flame retardancy of material. The data shows that UHMWPE was ignited quickly (*t*_ign_ = 39 s), the HRR reaches a peak value of 745.2 kW m^−2^ at 190 s, and basically no char layer remained after the test. Compared with UHMWPE, the *t*_ign_ of UHMWPE composites increases remarkable which gives more safe time in the fire accident, whereas the *t*_PHRR_ of UHMWPE composites decrease in little. Compared with UHMWPE and UHMWPE/(APP/EG)20, the UHMWPE/(APP/EG@PDA)20 composite display 59.5% and 71.5% reduction in the PHRR (Fig. 5a), 13.1% and 30.9% reduction in THR (Fig. 5b), respectively. The results confirm that EG@PDA can effectively decrease the heat release of UHMWPE composites.

**Table 6:** Parameters from cone calorimeter tests

Samples	*t*_ign_ (s)	*t*_PHRR_ (s)	PHRR (kW m^−2^)	THR (MJ m^−2^)	TSR (m^2^ m^−2^)	Residual weight (%)
UHMWPE	39	190	745.2	125	1265.5	0.06
UHMWPE/(APP/EG)20	85	175	301.7	108.6	1178.5	25.4
UHMWPE/(APP/EG@PDA)20	84	160	212.2	86.4	597.1	44.4
UHMWPE/APP20	111	240	504.5	128.3	1893.1	16.3

As shown in Fig. 5c, UHMWPE/APP20 gives higher TSR values than pure UHMWPE, indicating that the introduction of APP in UHMWPE leads to high smoke emission.^30^ Moreover, UHMWPE/APP15, UHMWPE/(APP/EG)15 and UHMWPE/(APP/EG@PDA)15 give a decrement order in TSR value, revealing that EG have obviously effect in suppressing product of smoke, even more PDA has better results in higher smoke suppress property.^31^

On the other hand, residual weight (Fig. 5d) of composites with flame retardants show an increase to some extent in comparison with the pure UHMWPE. And the UHMWPE/(APP/EG@PDA)20 obtains the highest rate of residual weight of all, indicating that the introduction of PDA could promote the formation of residual char. The more residual char at high temperature could lead to the better flame retardancy of the materials.^32^ Moreover, the formation of more char residue could effectively reduce the release of smoke particulates, resulting in low TSR.^33^

### 3.3. Thermal stability of the composites

**Fig. 6:**
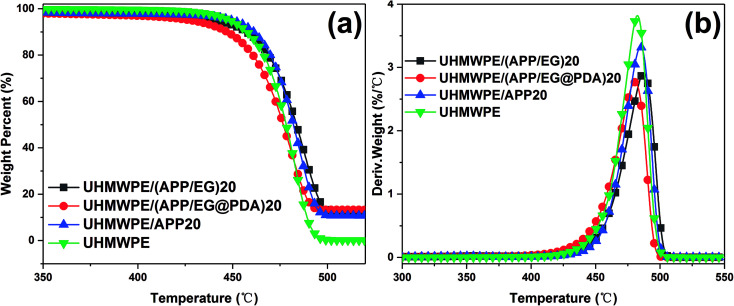
TGA and DTG curves of UHMWPE and its composite under N_2_ atmosphere.

The TGA and DTG curves and the calculated parameters of UHMWPE, UHMWPE/(APP/EG)20, UHMWPE/(APP/EG@PDA)20 and UHMWPE/APP20 composites are presented in Fig. 6 and Table 4. It is clear seen that the thermal degradation of pure UHMWPE in N_2_ atmosphere is characterized with *T*_5%_ and *T*_p_ at 448.4 °C and 484.2 °C, respectively. The char residue at 600 °C is about 0 wt%. As for UHMWPE/APP20, decomposition of beginning at the temperature (*T*_5%_) of 449.6 °C and maximum mass loss temperature (*T*_p_) at about 484.9 °C give slightly higher value compared with UHMWPE, indicating that the introduction of APP could promote thermal stability of UHMWPE. As for UHMWPE/(APP/EG)20, the lower *T*_5%_ and *T*_p_ in comparison with UHMWPE/APP20 shows that EG could decompose before the decomposition of UHMWPE. While for UHMWPE/(APP/EG@PDA)20, *T*_5%_ and *T*_p_ at 427.8 °C and 480.6 °C suggested that the utilization of PDA could decrease the thermal stability of UHMWPE composite because of the decomposition of PDA. This may be attributed to that PDA could decompose to gas and free radical to flame retardant UHMWPE, and EG decompose to H_2_SO_4_ to protect UHMWPE in condense phase. In addition, the UHMWPE/(APP/EG@PDA)20 gives highest value at 13.1% of residual weight of all composites indicates that the enhancement of flame retardancy by incorporation of PDA.

### 3.4. Char residue analysis

**Fig. 7:**
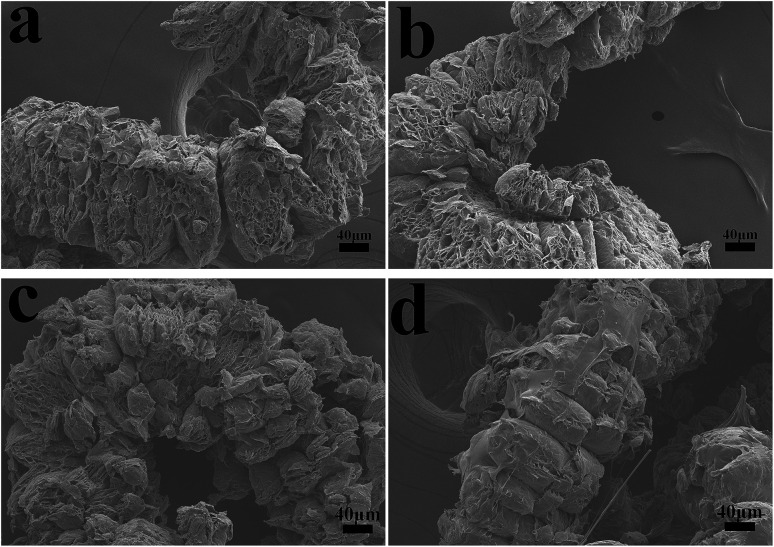
SEM micrographs of residual chars for UHMWPE/(APP/EG)20: (a) internal surface, (b) external surface and UHMWPE/(APP/EG@PDA)20: (c) internal surface, (d) external surface.

**Fig. 8:**
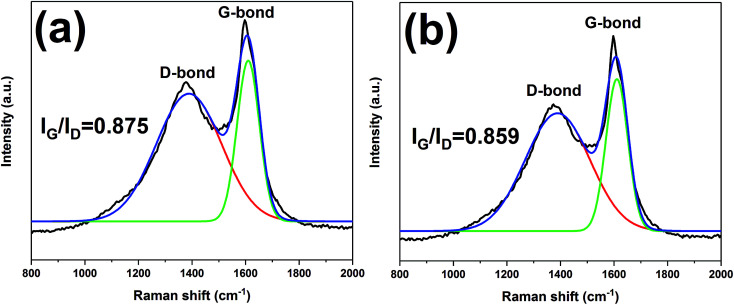
Raman spectra of the char residues of UHMWPE/(APP/EG)20 (a) and UHMWPE/(APP/EG@PDA)20 (b).

The char residues of UHMWPE composites from cone calorimeter test were examined by SEM analysis. The results are given in Fig. 7. In Fig. 7a and b, both internal and external surfaces of the char residue from UHMWPE/(APP/EG)20 exhibits a loose, discontinuous and brittle char layer with relatively large interlayer gaps. This will lead to UHMWPE with continuous flame which transfer to the material below. However, in Fig. 7c and d, the charred residue of UHMWPE/(APP/EG@PDA)20 gives a compact, continuous, and integrated char layer with seldom holes. This kind of char layer is suggested to act as an efficient and good barrier to prevent the combustible gas and heat flow from transferring into the UHMWPE. This may be attributed to scavenge free radical by the degradation of PDA can effectively agglomerate the carbon layer at high temperature.^18,32^ It easily can be seen that polydopamine may react with the degraded behaviors of UHMWPE composites, which forms a crosslinked char structure. Besides, Raman spectroscopy was used to determine the degree of graphitization of char residue.^34^ The intensity ratio of G-band to D-band (*I*_G_/*I*_D_) was applied to directly detect the degree of graphitization (Fig. 8).^25^ Compared with *I*_G_/*I*_D_ value of 0.875 for UHMWPE/(APP/EG)20 residual char, the residual char of UHMWPE/(APP/EG@PDA)20 possess lower *I*_G_/*I*_D_ value of 0.859. This result may attribute to the crosslinking role of degraded compound of PDA in residual char, which destroys the crystalline graphite. As a result, PDA could benefit to the formation of compact and crosslink structure of residual char. In addition, PDA has a good effect to scavenge the free radicals in the combustion process.^19^ The radicals scavenging activity can act in the gas phase, which leads to the suppression of combustion and degradation of UHMWPE composites.

### 3.5. Flame retardant mechanism analysis

**Fig. 9:**
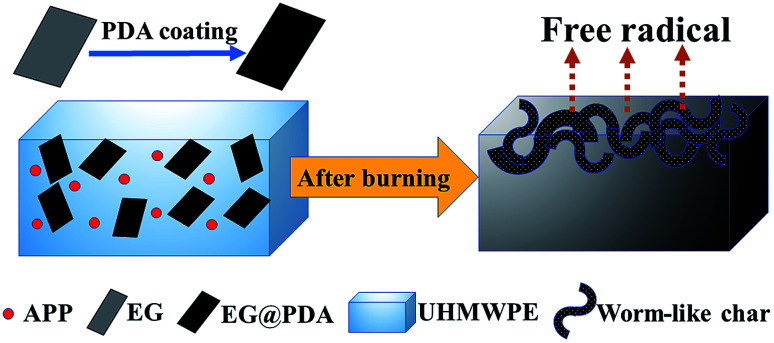
Schematic diagram of flame-retardant mechanism.

Schematic combustion processes of UHMWPE/(APP/EG@PDA) composites are illustrated in Fig. 9. Based on the before discussion, it can be concluded that EG@PDA can effectively retard the thermal degradation of UHMWPE due to EG@PDA can decompose to gas phase to scavenge free radical and form compact structure under high temperature. It can be seen that EG@PDA and APP are randomly dispersed in the UHMWPE matrix. EG@PDA is the PDA coated EG. Randomly dispersed APP didn't react with EG@PDA. After burning, owing to the presence of EG, UHMWPE composite decompose to many constructions of worm-like which acts as a physical barrier for suppression of heat and gas transfer. The presence of APP could decompose and release NH_3_ to help construction of barrier of worm-like structure. Also, PDA would pre-decompose before UHMWPE and release reactive group to react with free radical in the combustion process. The degraded resultant of PDA could scavenge free radical to retard flame in combustion of UHMWPE.

### 3.6. Mechanical properties of the UHMWPE composites

**Fig. 10:**
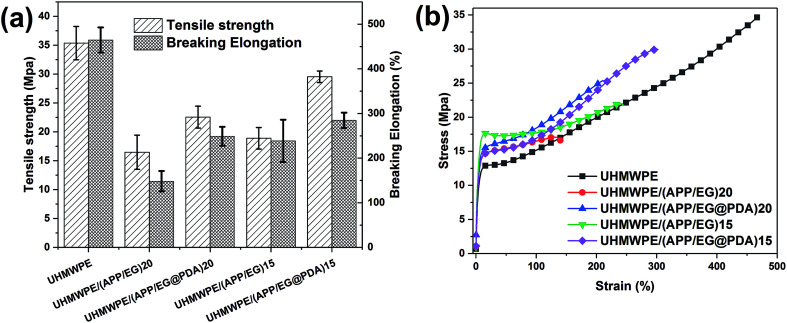
Tensile strength and elongation at break histogram and the strain–stress curve of UHMWPE composites.

Table 7 and Fig. 10 provides the mechanical properties of UHMWPE and UHMWPE composites. As shown in Table 7 and Fig. 10, the tensile strength and elongation at break of UHMWPE are 35.36 MPa and 464.68% respectively. Adding inorganic particles (APP, EG or EG@PDA) to UHMWPE will lead to a significant deterioration of its mechanical properties. However, compared with UHMWPE/(APP/EG)20, UHMWPE/(APP/EG@PDA)20 possesses 22.57 MPa in the tensile strength and 248.93% in the elongation at break, which increases by 37% and 67.9%, respectively. Besides, UHMWPE/(APP/EG@PDA)15 has 29.54 MPa in the tensile strength and 284.66% in the elongation at break, which increases by 56.3% and 19.2% compared to UHMWPE/(APP/EG)15. These results consistent with stress–strain curves in Fig. 10b suggest that the introduction of EG@PDA to UHMWPE largely enhance the mechanical properties of UHMWPE composites.

**Table 7:** The mechanical properties of UHMWPE and its flame-retardant composites

Samples	Tensile strength (MPa)	Elongation at break (%)
UHMWPE	35.36 ± 2.89	464.68 ± 28.23
UHMWPE/(APP/EG)20	16.47 ± 2.97	148.28 ± 22.84
UHMWPE/(APP/EG@PDA)20	22.57 ± 1.9	248.93 ± 21.22
UHMWPE/(APP/EG)15	18.9 ± 1.87	238.72 ± 47.19
UHMWPE/(APP/EG@PDA)15	29.54 ± 0.99	284.66 ± 17.17

**Fig. 11:**
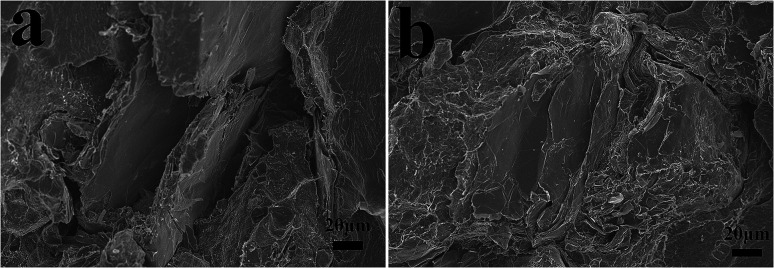
The fracture morphology of (a) UHMWPE/(APP/EG)20 and (b) UHMWPE/(APP/EG@PDA)20.

Fig. 11 can observe the interfacial adhesion of UHMWPE/(APP/EG)20 and UHMWPE/(APP/EG@PDA)20 from the fracture surface in tensile test. For UHMWPE/(APP/EG)20, obvious gaps indicate poor interfacial compatibility between the EG and UHMWPE matrix. However, for UHMWPE/(APP/EG@PDA)20, EG coated with PDA has no distinct gaps between EG@PDA and the UHMWPE matrix, suggests a good interfacial compatibility. The PDA can improve the compatibility between the UHMWPE matrix and EG@PDA since the produced hydrogen bond could connect with UHMWPE. Therefore, the method of EG coated PDA is a good way to enhance mechanical properties.

## 4. Conclusions

PDA coated EG (EG@PDA) were successfully prepared through the surface treatment process. The resultant EG@PDA can significantly improve the flame retardancy and mechanical property for UHMWPE. Specifically, the incorporation of EG@PDA into UHMWPE can reduce the flammability (including PHRR, THR, LOI, *etc.*). It is interesting to note that EG@PDA can not only be used as a multifunctional modifier to improve the thermal stability and mechanical property, but also can act as an efficient flame retardant and smoke suppressant for UHMWPE. This may be attributed to the degraded compound of PDA can scavenge the free radical and form the crosslink structure of residual char to improve the effectiveness of flame retardancy at high temperature.

## Conflicts of interest

There are no conflicts to declare.

## Supplementary Material

## References

[cit1] Araby S., Wang C. H., Hao W., Meng Q., Kuan H. C., Kim N. K., Mouritz A., Ma J. (2018). Composites, Part A.

[cit2] Luo F., Wu K., Wang S., Lu M. (2017). Compos. Sci. Technol..

[cit3] Maksimkin A. V., Danilov V. D., Senatov F. S., Olifirov L. K., Kaloshkin S. D. (2017). Wear.

[cit4] Chih A., Ansón-Casaos A., Puértolas J. A. (2017). Tribol. Int..

[cit5] Brockett C. L., Carbone S., Fisher J., Jennings L. M. (2017). Wear.

[cit6] Korobeinichev O. P., Gonchikzhapov M. B., Paletsky A. A., Tereshchenko A. G., Shundrina I. K., Kuibida L. V., Shmakov A. G., Hu Y. (2017). Proc. Combust. Inst..

[cit7] Hou S., Yong J. Z., Jiang P. (2018). Polym. Degrad. Stab..

[cit8] Luo F., Wu K., Lu M., Nie S., Li X., Guan X. (2015). J. Therm. Anal. Calorim..

[cit9] Zhu Z. M., Rao W. H., Kang A. H., Liao W., Wang Y. Z. (2018). Polym. Degrad. Stab..

[cit10] Zheng Z., Liu Y., Zhang L., Wang H. (2016). J. Mater. Sci..

[cit11] Tang M., Fei Q., Man C., Sun Z., Yang X., Chen X., Shen Z. Z. A. (2016). Polym. Adv. Technol..

[cit12] Wang H., Cao J., Cao C., Guo Y., Luo F., Qian Q., Huang B., Xiao L., Chen Q. (2018). Polym. Adv. Technol..

[cit13] Huang J., Tang Q., Liao W., Wang G., Wei W., Li C. (2017). Ind. Eng. Chem. Res..

[cit14] Lee H., Dellatore S. M., Miller W. M., Messersmith P. B. (2007). Science.

[cit15] Ju K.-Y., Lee Y., Lee S., Park S. B., Lee J.-K. (2011). Biomacromolecules.

[cit16] Zhang L., Li Z., Pan Y.-T., Yáñez A. P., Hu S., Zhang X.-Q., Wang R., Wang D.-Y. (2018). Composites, Part B.

[cit17] Cao J., Wang H., Cao C., Li H., Xiao L., Qian Q., Huang B., Chen Q. (2019). Polym. Int..

[cit18] Luo F., Wu K., Shi J., Du X., Li X., Yang L., Lu M. (2017). J. Mater. Chem. A.

[cit19] Cho J. H., Vasagar V., Shanmuganathan K., Jones A. R., Nazarenko S., Ellison C. J. (2016). Chem. Mater..

[cit20] Cai W., Guo W., Pan Y., Wang J., Mu X., Feng X., Yuan B., Wang B., Hu Y. (2018). Composites, Part A.

[cit21] Lee W., Lee J. U., Jung B. M., Byun J. H., Yi J. W., Lee S. B., Kim B. S. (2013). Carbon.

[cit22] Missale C., Nash S. R., Robinson S. W., Jaber M., Caron M. G. (1998). Physiol. Rev..

[cit23] Wise R. A. (2004). Nat. Rev. Neurosci..

[cit24] Perret B., Schartela B., Ciesielski M., Diederichs J., Döring M., Krämer J., Altstädt V. (2011). Eur. Polym. J..

[cit25] Cai W., Wang J., Pan Y., Guo W., Mu X., Feng X., Yuan B., Wang X., Hu Y. (2018). J. Hazard. Mater..

[cit26] Cui M., Ren S., Zhao H., Xue Q., Wang L. (2018). Chem. Eng. J..

[cit27] Xiang Y., Pang Y., Jiang X., Huang J., Xi F., Liu J. (2018). Appl. Surf. Sci..

[cit28] QUN XU L. I., Wen J. Y., Koon-Gee N., En-Tang K., Guo D. F. (2010). Macromolecules.

[cit29] Shen L., Li J., Lin H., Feng S., Zhang Y. (2017). Polym. Bull..

[cit30] Tang M., Qi F., Chen M., Sun Z., Xu Y., Chen X., Zhang Z., Shen R. (2016). Polym. Adv. Technol..

[cit31] Wang J., Wang H., Geng G. (2018). J. Colloid Interface Sci..

[cit32] Zhu M., Muhammad Y., Hu P., Wang B., Wu Y., Sun X., Tong Z., Zhao Z. (2018). Appl. Catal., B.

[cit33] Yan L., Xu Z., Wang X. (2017). Prog. Org. Coat..

[cit34] Marc S., Iso C., Scheinost A. C., Chris J., Ruben K. (2005). Environ. Sci. Technol..

